# A Case of Injury of the Eye

**Published:** 1847-08

**Authors:** Chs. W. Stephens

**Affiliations:** St. Louis


8. *A Case of Injury of the Eye.* By Chs. W. Stephens, M.D., St. Louis.—*April* 15—Mr. Newell a clerk in a drug store, in combining eight ounces of sulphuric acid with two ounces of Indigo, imprudently corked the bottle containg the ingredients, and upon effervescence taking place, the cork was forced out, and the contents received upon the face and into the eyes of the gentleman.

Dr. Jackson and myself visited him in about ten minutes after the accident, and, at the suggestion of the doctor, rubbed the face with the calcined magnesia, at the same time raising the lids, deposited small quantities upon the globe of the eye. The effect of this application was immediately to neutralize the acid, forming the sulphate of magnesia, which is inert. High inflammation immediately supervened, of conjunctivæ and of the whole face; applied a mixtnre of the cal. magnesia with olive oil, and poultices of slippery elm; the inflammation soon began to subside, and we directed an astringent collyrium. He is now (May 10) able to attend to his usual business, suffering, however, a good deal from an inversion of the eye-lashes, *trichiasis*, which I find it necessary to remove from time to time; the lower lachrymal duct is entirely obliterated.—*Missouri Med. and Surg. Jour.*

